# Stamp Approach for Posterior Composite Restorations: A Case Report

**DOI:** 10.7759/cureus.26584

**Published:** 2022-07-05

**Authors:** Paridhi Agrawal, Pradnya Nikhade

**Affiliations:** 1 Dentistry, Sharad Pawar Dental College And Hospital, Datta Meghe Institute of Medical Sciences, Wardha, IND

**Keywords:** bio-mimetic composite restoration, aesthetic composite restoration, class i restoration, posterior composite restoration, stamp technique

## Abstract

Considering the esthetic and deleterious effects of silver amalgam restorations, the preferred filling material for posterior restoration is now the composites. The novel stamping procedure can be considered as a negative reproduction or small imprint of the occlusal tooth topography, which can be utilized in instances when there is no visible cavitation or tooth structural loss but radiographic evidence of caries. Newer approaches are being developed to overcome previous issues and shorten the time required. When the tooth's pre-operative anatomy remains intact and not damaged by caries, the stamp technique uniquely simulates the original and natural occlusal topography of teeth using composite restorations. Here, an occlusal matrix is created from a tooth's unhindered occlusal surface to replicate a precise tooth-like restoration. Consequently, it helps restore the tooth's natural contact and contour while maintaining an accurate functional occlusion. Furthermore, the amount of time spent completing and polishing is minimized. The stamping technique is a one-of-a-kind, biomimetic process for restoring class I cavities that replicate the tooth's identical morphology.

## Introduction

The amalgam period is passed, and more aesthetically acceptable restorations have taken their place. Amalgam has many drawbacks, including mercury poisoning and being non-esthetic. Nowadays, posterior composite resin restorations are the standard in modern dentistry, which is moving towards Bio-mimetic dentistry. Bio-mimetic translates to mimicking nature [1]. In addition, the earlier prophylactic concept of extension is being displaced by minimally invasive dentistry, which focuses on preserving the sound dental structure.

Although dentists increasingly use composite restorations and handcrafting, a beautiful direct composite repair requires expertise and accuracy. Direct restorations can be technique-dependent and do not always result in an exact replication of tooth shape and occlusion. In addition, as compared to amalgam restorations, the time required for finishing and polishing the restoration is doubled [2]. Myrahin et al. invented a revolutionary method called the Stamp technique to combine function and aesthetics [3].

This novel stamping procedure begins with creating an index, which can be considered a negative reproduction or small imprint of the occlusal topography. It can be utilized when there is no visible cavitation or tooth structural loss but radiographic evidence of caries. This index is placed against the final composite increment before curing, yielding a positive imitation. The pre-existing state is reproduced, with the added benefit of less time spent removing excess and polishing repairs [4].

## Case presentation

**Figure 1:**
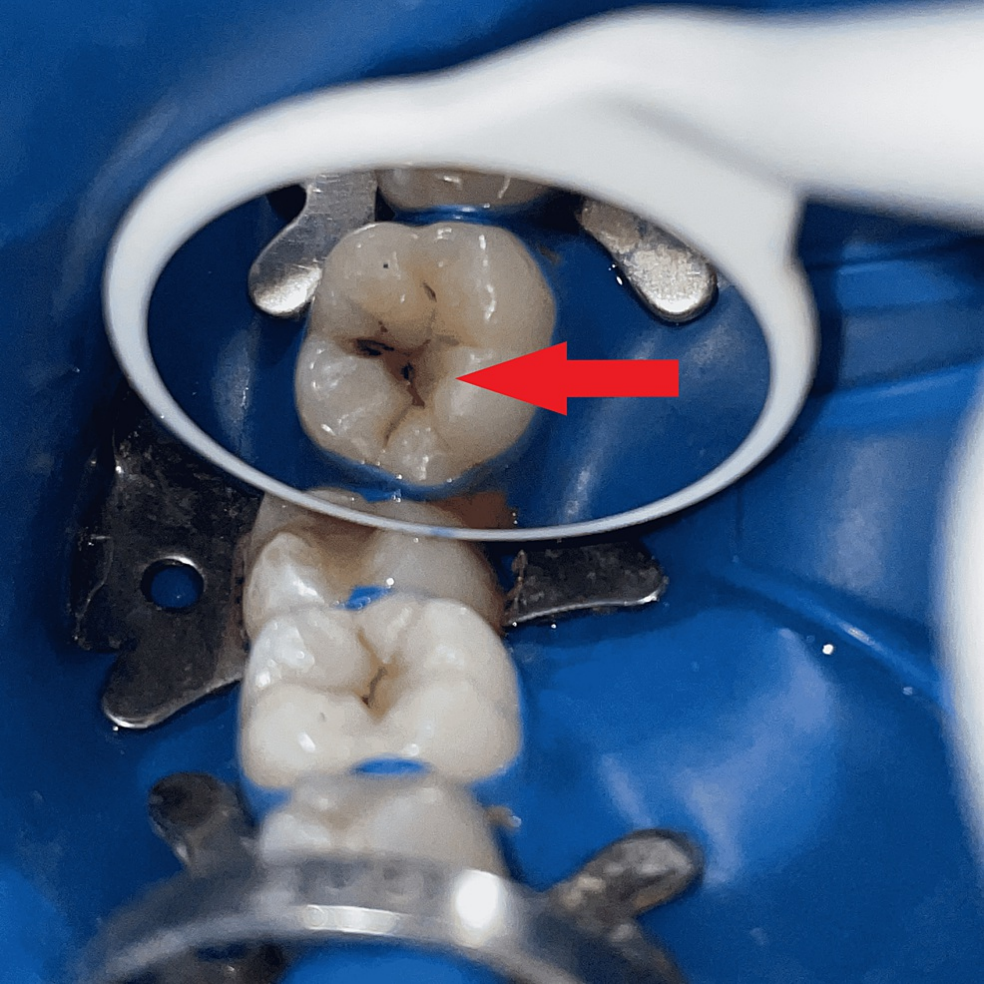
Pre-operative photograph

A 23-year-old male patient reported to the Department of Conservative Dentistry and Endodontics and complained of black stains on his lower left back tooth region (Figure 1). There was no relevant medical history to be found. An intraoral examination revealed good oral hygiene with pit and fissure caries concerning 36. An intraoral periapical radiograph was used to determine the extent of caries, and the marginal ridge was not involved. Consequently, it was decided to employ composite resin and a stamp method to rebuild the decayed area.

Treatment

**Figure 2:**
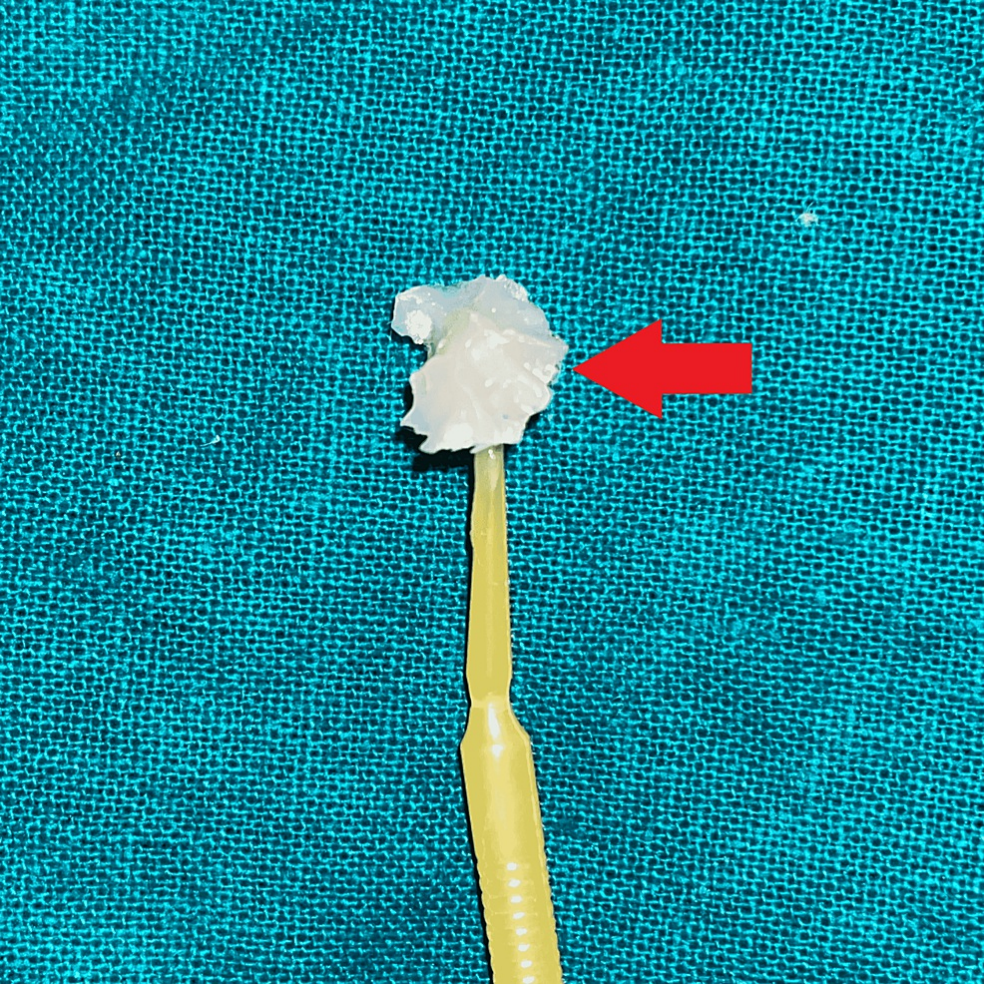
Obtained stamp

**Figure 3:**
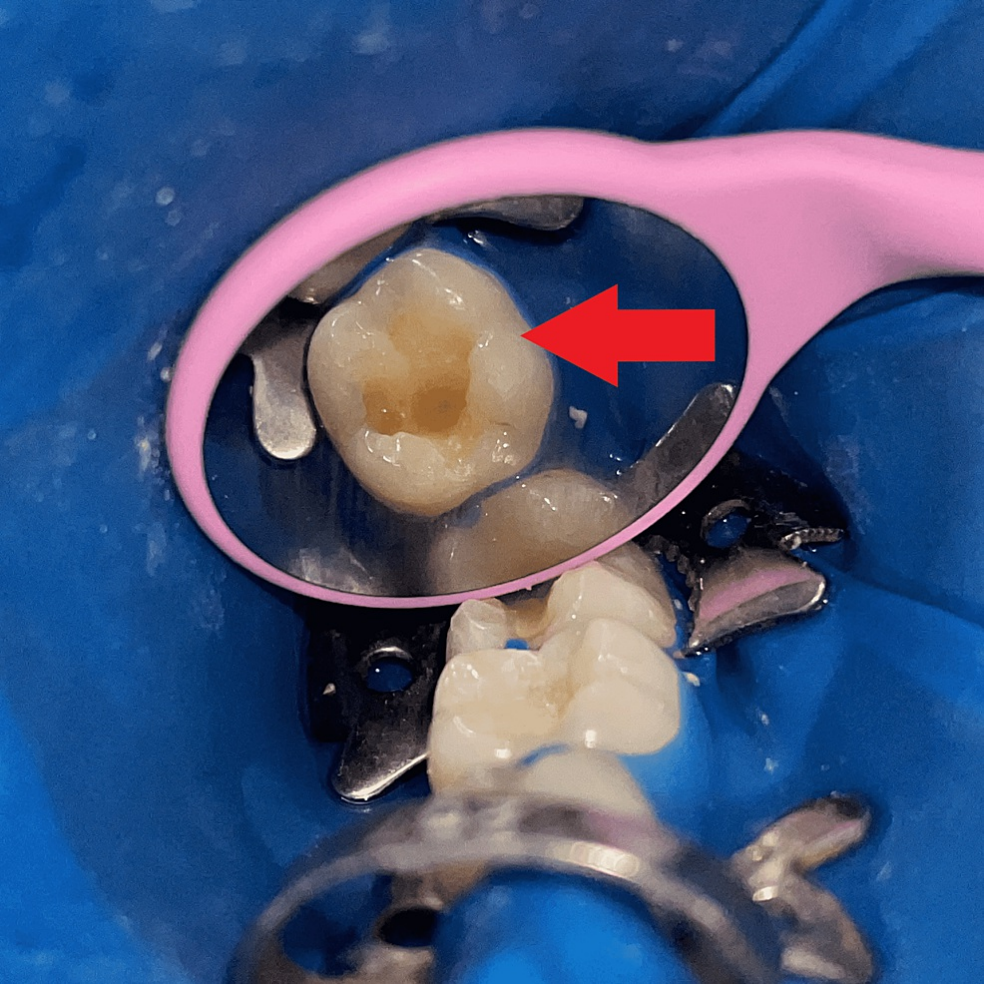
Caries excavation and cavity preparation

The tooth was isolated with a rubber dam (GDC Dental dam kit, India), then separating media (Zartex, Zarir & Zaida Industries, Malaysia) was applied with a brush on the tooth surface. A stamp was created by applying flowable composite (Ivoclar Tetric N-Flow, USA) on the tooth's intact occlusal surface. To make the stamp, a micro brush tip was cut to function as a handle and dipped in composite, followed by polymerization by light-curing (Figure 2). The carious lesion was totally excised, and a class I cavity was created (Figure 3).

**Figure 4:**
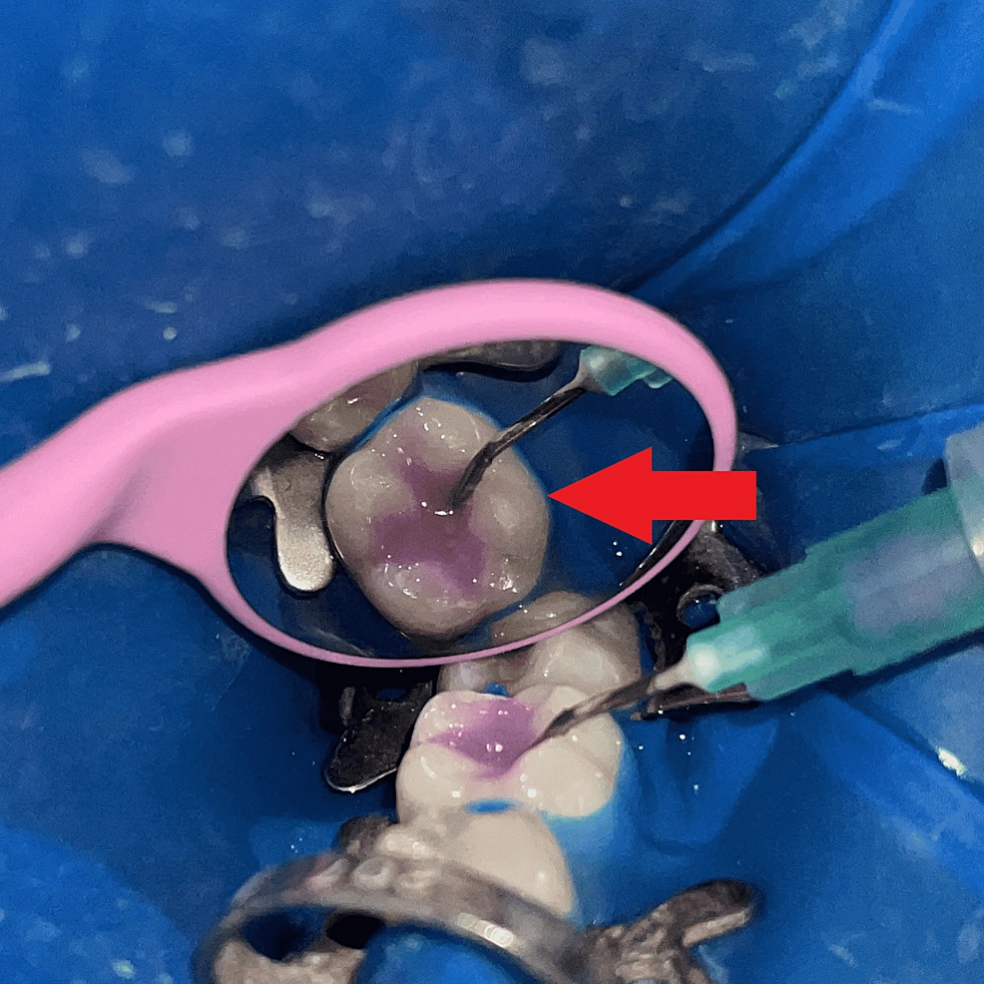
Etching with 37% Phosphoric acid

**Figure 5:**
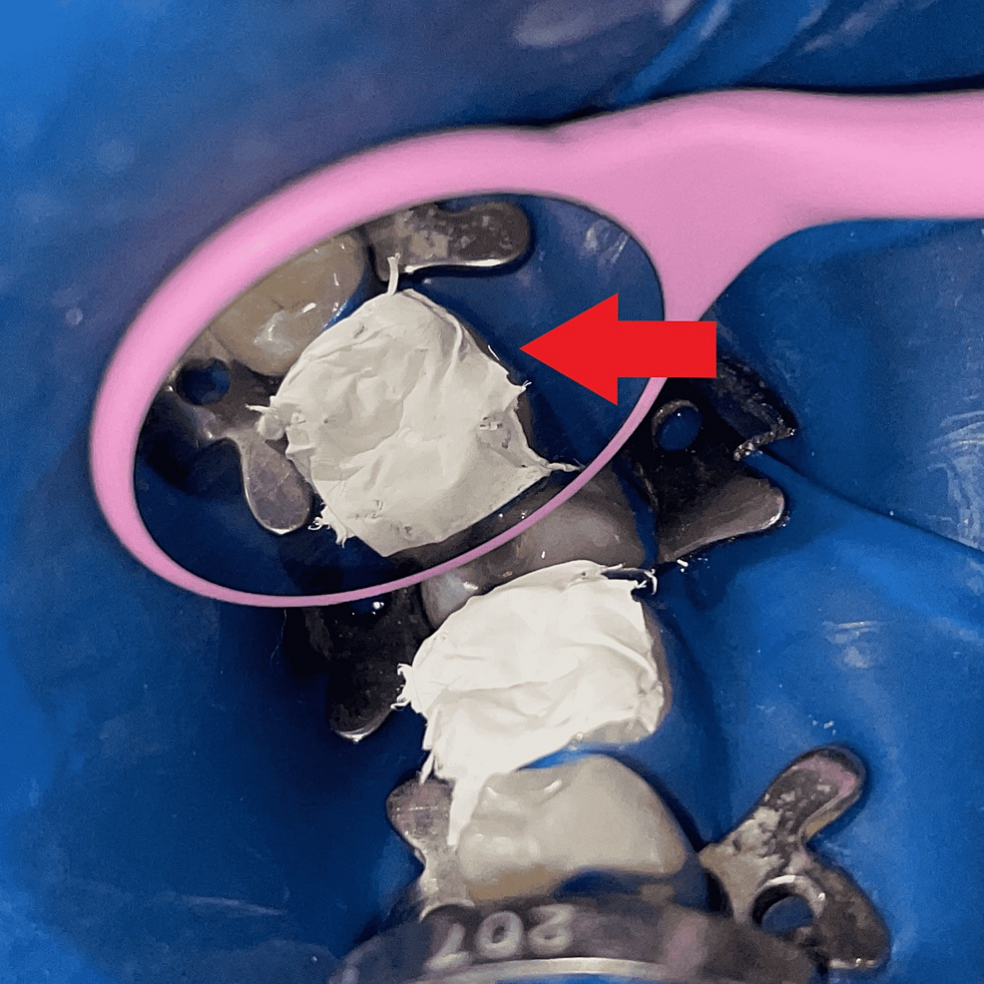
Teflon application

**Figure 6:**
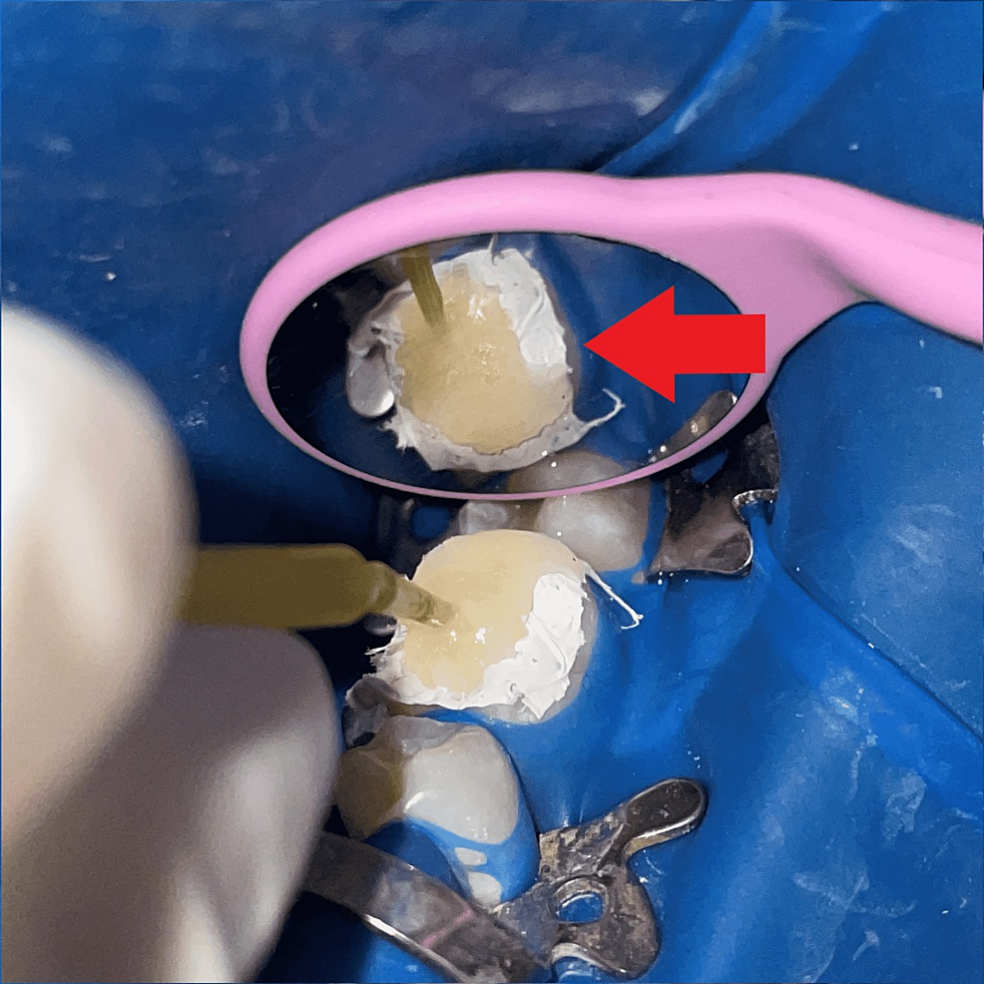
Stamp placement

**Figure 7:**
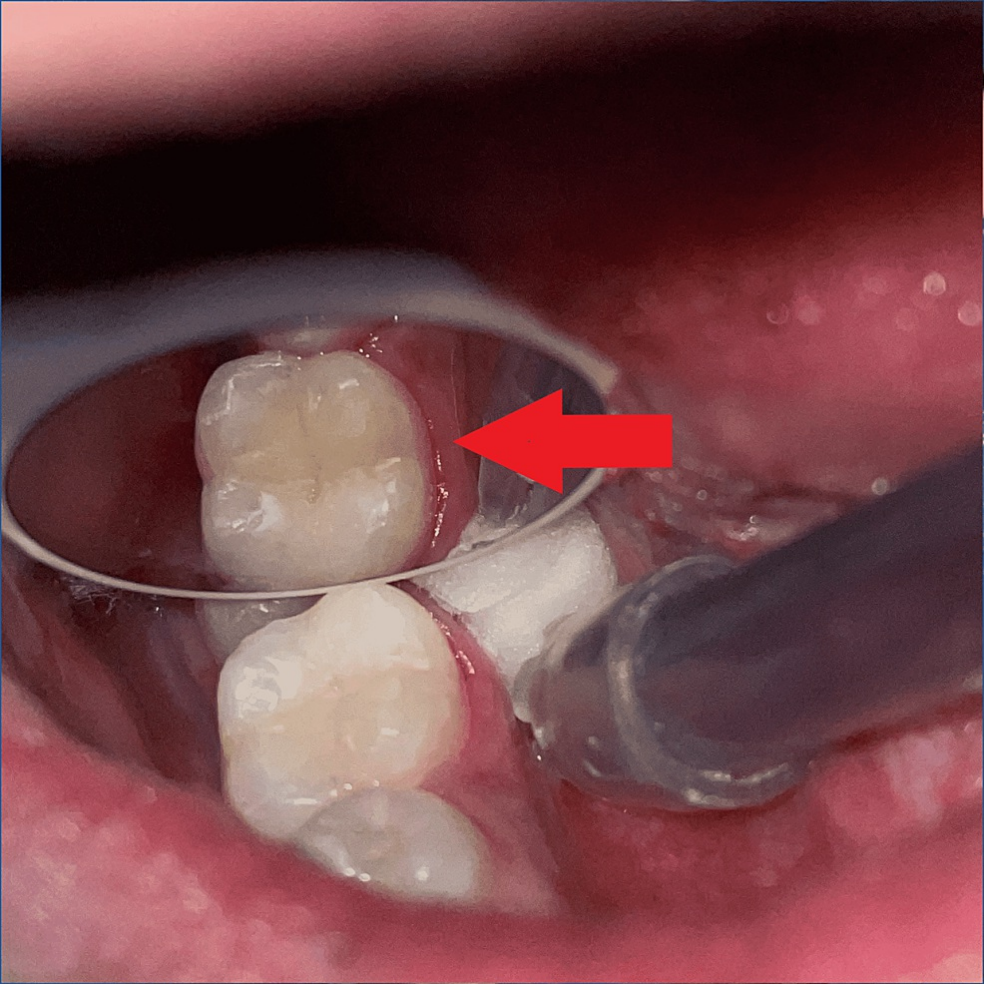
Postoperative photograph

Etching (Figure 4) was done for 30 seconds using 37% orthophosphoric acid (Prime dental, India), then rinsed and air-dried with a 3-way syringe. The bonding agent (3m ESPE Single Bond Universal, USA) was applied and light-cured for 20 seconds. The cavity was gradually restored with composite (Spectrum Dentsply Sirona, USA) up to 1mm below the occlusal surface, with light-curing for 20 seconds. Before curing, the last layer of composite was applied, and a piece of Teflon tape was affixed to the occlusal surface (Figure 5). After that, the micro brush occlusal stamp was placed over the tape and gradually pressed with light pressure (Figure 6). The extra material was removed, and the composite was polymerized. Minimal finishing and polishing were done using a polishing paper disc (Shofu-snap Mini snap kit, Kyoto, Japan) (Figure 7).

Outcome and follow-up

The patient received instructions on postoperative care, and regular follow-up was advised yearly.

## Discussion

Any restoration's ultimate objective is to restore normal form and function while also making dental treatment more comfortable for the patient. This case aids in achieving the aim above by maintaining a harmonious cusp fossa relationship with the antagonist and neighboring teeth. When the tooth structure is intact but has caries beneath it, this treatment is utilized (class I and class II). Cling film can be used instead of Teflon tape since it does not need to be removed during curing. It also shields composite materials from numerous exposures to ambient light [5].

The key benefit of this approach is that, if mastered, it may require less overall time due to the immediate cusp fossa relation. This is beneficial to the practitioners and helps to strengthen their reputation among patients [6]. In addition, the degree of porosity in the final restoration is significantly decreased. It is because the stamped matrix exerts pressure on the composite, reducing the production of microbubbles and preventing oxygen from interfering with the polymerization of the final layer of composites [4]. These characteristics have been demonstrated to be significant predictors for composites' long-term performance [7].

While the technique's downside is that it needs expertise and clinical knowledge to conduct it properly, the approach is cost-ineffective because it needs the use of a micro brush and flowable composite, both of which are costly. Expired flowable composite or clear acrylic resin can be utilized to solve this disadvantage [8].

Composite resin restorations can be placed using a variety of methods. Both direct and indirect methods are used. Choosing between direct and indirect techniques is a difficult decision to make. The preservation of tooth structure is possible with single-visit direct posterior restorations [9]. The benefits of the direct approach are the improved strength of the remaining tooth structure and the possibility of repair. However, these restorations' mechanical strength is less when compared to indirect restorations. In addition, there are several drawbacks, such as proximal and occlusal wear, surface roughness, marginal discoloration, cusp flexure, loss of marginal integrity, secondary caries, postoperative sensitivity, method sensitivity, and poor bonding to dentin, and low fracture toughness [10].

The term "indirect technique" describes the process of fabricating a restoration outside of the mouth in a laboratory. Then with the help of resin cement, it is luted to the tooth [10]. Composite inlays increase wear and fracture resistance, decrease polymerization shrinkage, better contoured proximal surfaces, occlusal contacts, and biocompatibility [11]. The disadvantages of composite inlays are their increased cost and time requirements, the need for two appointments, the creation of a temporary restoration, and their low repairability [10]. Considering all the pros and cons of the stamp technique, keeping in mind the incremental placement of composite restoration to overcome polymerization shrinkage, the stamp technique improves workflow providing occlusal anatomy near perfection.

## Conclusions

Each restoration method, including traditional and stamp methods, has benefits and drawbacks. The benefit of the stamp technique is that it may achieve the precise anatomical shape of the tooth or biomimetic without the need for operator expertise. Also, occlusal topography has far more precision and accuracy than the manual approach. Along with that, it makes the polishing of the composite easier, cutting down on labor time. The drawback of this treatment is that it is only recommended for caries with an occlusal architecture that is still intact, such as pit and fissure caries or cavities at the margins with intact occlusal surfaces. The conventional method can be used on relatively large, atypically shaped caries that may cause greater composite shrinkage during polymerization, but it needs operator expertise and takes longer.
